# CO_2_ and O_2_ solubility and diffusivity data in food products stored in data warehouse structured by ontology

**DOI:** 10.1016/j.dib.2016.04.044

**Published:** 2016-04-26

**Authors:** Valérie Guillard, Patrice Buche, Juliette Dibie, Stéphane Dervaux, Filippo Acerbi, Estelle Chaix, Nathalie Gontard, Carole Guillaume

**Affiliations:** a UMR IATE, University of Montpellier – INRA, 2 place Pierre Viala, F-34060 Montpellier Cedex 1, France; b AgroParisTech & INRA UMR MIA 518, 16, rue Claude Bernard, F-75 231 Paris Cedex 05, France

**Keywords:** Diffusivity, Solubility, Data, Data warehouse, Ontology, Food, O_2_, CO_2_

## Abstract

This data article contains values of oxygen and carbon dioxide solubility and diffusivity measured in various model and real food products. These data are stored in a public repository structured by ontology. These data can be retrieved through the @Web tool, a user-friendly interface to capitalise and query data. The @Web tool is accessible online at http://pfl.grignon.inra.fr/atWeb/.

**Specifications Table**

**Table:** 

Subject area	*Biochemistry*
More specific subject area	*Food science and food engineering*
Type of data	*Table, links*
How data was acquired	*Chemical titration (for CO*_*2*_*quantification) and luminescence-based detection (for O*_*2*_*detection) implemented in dedicated experimental set-ups*
Data format	*Analyzed, ready to use*
Experimental factors	*Samples considered are model and real food products without any pre-treatment except addition of sodium azide to avoid microbial growth*
Experimental features	*Solubility is measured by quantifying the concentration of dissolved gas in a sample in equilibrium with a fix and controlled partial pressure.*
*Diffusivity is identified from an experimental diffusion kinetic curve by using a mathematical model and appropriate numerical treatment (algorithm of optimization).*
Data source location	*University of Montpellier, FR-34060, France*
Data accessibility	*Data is within this article.*

**Value of the data**

• A unique set of CO_2_ solubility and diffusivity data indispensable in food engineering to model CO_2_ gas transfer in food.
• A unique set of O_2_ diffusivity values within synthetic oils as a function of temperature.
• O_2_ diffusivity data could be used to predict oxidation of O_2_-sensitive compounds in foods.
• These data could serve as benchmark for other researchers coping with research on gas transfer in food for numerous simulation.

## 1 Data

Data shared with this article are more than 100 data of solubility and diffusivity of gases (O_2_ and CO_2_) in food samples. These data are stored in a data warehouse called @Web in which the data management is guided by ontology.

All data are available for uploading at the URL specified below and recalled in the table hereafter with the details about the nature and amount of data available at each URL.

**Table:** 

**Data type**	**Table URL (copy/paste the URL in your Internet browser)**	**Amount of data**
CO_2_ solubility	*pfl.grignon.inra.fr/atWeb/TableServlet?viewTable=2775&idDoc=1335&id=35272672*	34
*pfl.grignon.inra.fr/atWeb/TableServlet?viewTable=2776&idDoc=1335&id=35305550*	21
*pfl.grignon.inra.fr/atWeb/TableServlet?viewTable=2773&idDoc=1335&id=35245144*	48
*pfl.grignon.inra.fr/atWeb/TableServlet?viewTable=2732&idDoc=1332&id=34354344*	3
CO_2_ diffusivity	*pfl.grignon.inra.fr/atWeb/TableServlet?viewTable=2780&idDoc=1346&id=35361064*	12
*pfl.grignon.inra.fr/atWeb/TableServlet?viewTable=2826&idDoc=1346&id=36350532*	11
*pfl.grignon.inra.fr/atWeb/TableServlet?viewTable=2779&idDoc=1346&id=35346176*	11
*pfl.grignon.inra.fr/atWeb/TableServlet?viewTable=2778&idDoc=1346&id=35333312*	12
*pfl.grignon.inra.fr/atWeb/TableServlet?viewTable=2777&idDoc=1346&id=35320430*	16
*pfl.grignon.inra.fr/atWeb/TableServlet?viewTable=2733&idDoc=1332&id=34367074*	11
O_2_ diffusivity	*pfl.grignon.inra.fr/atWeb/TableServlet?viewTable=2764&idDoc=1342&id=35103704*	24
*pfl.grignon.inra.fr/atWeb/TableServlet?viewTable=2755&idDoc=1342&id=34742706*	30
*pfl.grignon.inra.fr/atWeb/TableServlet?viewTable=2754&idDoc=1342&id=34730102*	10
*pfl.grignon.inra.fr/atWeb/TableServlet?viewTable=2765&idDoc=1342&id=35116534*	44
*pfl.grignon.inra.fr/atWeb/TableServlet?viewTable=2910&idDoc=1342&id=40225446*	2

## 2 Experimental design, materials and methods

**O**_**2**_. Oxygen optical sensors (Presens GmbH, Regensberg, Germany) were used to monitor O_2_ partial pressure. This measurement is based on dynamic luminescence quenching. Due to an excitation flash emitted through an optical fibre, the luminophore contained in the sensor goes into an excited state and thus emits fluorescence backscatter signal, which is detected by the optical fibre. If the luminophore is in contact with an oxygen molecule, the backscatter signal is changed due to a dynamic quenching of luminescence. The change in the backscatter signal permits to detect the O_2_ partial pressure in the medium. Two different set-ups exist (1) an invasive O_2_-sensitive optical sensor made of a syringe probe (micro-sensors, Presens GmbH, Regensburg, Germany) connected to the optical fibre and oxygen metre (Oxy-4 micro, Presens) and (2) a non-invasive oxygen sensor made of a dot of 5 mm of diameter that can be stuck on the wall of a transparent container and measurement is then made through the transparent container.

Oxygen sorption kinetics were measured at fixed temperature value when imposing a controlled partial pressure of O_2_ in the surrounding of the sample. The mono-directional O_2_ ingress into the sample was measured locally at the bottom or in the middle of the thin layer of food material previously free of O_2_ using one of the aforementioned sensors. More details on the experimental set-up could be found in [1], [2], [3].

**CO**_**2**_. The solubility of CO_2_ was measured at equilibrium by quantification of the gas dissolved in the sample using chemical titration [4], [5]. This measurement was done in a set-up where the sample is in a controlled chamber (controlled temperature, relative humidity, CO_2_ gas composition).

The diffusion of CO_2_ was characterised by (1) imposing a gradient of CO_2_ to a piece of material of simple geometry (cylinder or plane sheet), (2) measuring the CO_2_ sorption kinetic in the sample and (3) identifying diffusivity values by adjusting a dedicated mathematical model to the experimental kinetic. Two types of kinetic could be obtained: (1) CO_2_ space-dependent profile in the cylindrical sample after its slicing and CO_2_ quantification in each slice or (2) CO_2_ time-dependent profile after CO_2_ quantification in each thin slice (one slice corresponding at one time of kinetic) [4], [6].

Numerical treatment. For both O_2_ and CO_2_, diffusivities are identified by fitting a dedicated mathematical model to the experimental kinetic curve (space-dependent profile or time-dependent profile). This identification step is performed using a routine (“lsqnonlin”) of Matlab® software.
